# A call to predoctoral researchers: introducing the Robin Weiss Science Writing Prize—celebrating creativity in science

**DOI:** 10.1093/oxfimm/iqag012

**Published:** 2026-07-21

**Authors:** Daniel M Altmann

**Affiliations:** Department of Immunology and Inflammation, Imperial College London, London, W12 0NN, United Kingdom

What is it that medical journal editors actually do? Those who haven’t been closely involved with the inner workings of academic journals might imagine the role as essentially a gatekeeper, deciding which of the submitted manuscripts may or may not be published. If the journal is operating well with good pathways for requesting and evaluating peer review from relevant experts, the gatekeeper role is a fairly minor part of the job description. The major part is the over-arching need to bring added value to the science discipline, in our case infection and immunity, by offering the best possible forum for the community to share new ideas and move knowledge forward. The world of academic science publishing has spent recent years in a state of upheaval as we appraise transition from centuries of dusty, leather-bound tomes of paper journals on library shelves, to embrace the greater equity and immediacy of open-access, online publishing. This offers new vistas of what medical publishing can be—a hybrid, offering the best synthesis between the gravitas of the old manuscripts combined with the impact and immediacy of social media posts or chatroom debates. Yet, at the heart of this, the challenge to the researcher remains the same: the remit is to have an important, novel research question, conduct some incisive research that will answer the question or at least advance understanding of it, then write about it for publication in a way that is accessible, readable and persuasive. No matter how talented in the lab, we are all only as good as our ability to write it down, publish, and spread the word. As research scientists or doctors, we receive little or any training in this last part—how to write about our findings clearly and beautifully—yet this is just as critical a part of the job as knowing how to use a pipette or run a stats analysis. These were some of the considerations that led to Oxford University Press inaugurating the Robin Weiss Science Writing Prize, a scheme to promote and catalyse good writing in early stage researchers (https://academic.oup.com/ooim/pages/robin-weiss-prize).

Robin, who died earlier this year, was a truly exemplary role model in terms of the composite skillset of the ultimate scientist: broad background knowledge and intellectual curiosity as a platform from which to ask important questions, the technical wherewithal to conduct robust and informative experiments, and then, the joy of writing about it creatively, beautifully and convincingly so that new ideas become accessible. His scientific career has been described in detail elsewhere [1, 2]. The huge achievements spanned transformative discoveries in endogenous retroviruses (which initially met with scepticism but are now appreciated to comprise about 10% of the human genome), viral oncogenes, and HIV retrovirology including the description of CD4 as the HIV receptor and development of the first antibody tests for HIV. He was also a warm-spirited, generous contributor to the international research community, whether through his roles on funding panels or his training of a generation of the most gifted scientists at the interface of virology, oncology and immunology. All of these are part of his enduring legacy. A special part of this legacy is the creativity and eclecticism of his science writing. This was a man of encyclopaedic knowledge and boundless curiosity, whose passion for discovery shines through in all that he wrote. There are many quotable examples. This is from the opening of an early 2000s piece for Nature Medicine where Weiss was asked to reflect on the next 20-years of HIV-AIDS research [3]: ‘Apollo conferred on Cassandra the gift of clairvoyance but later added the caveat that no one would listen to her prophesy….Our colleagues at the Institut Pasteur must have felt as frustrated as Cassandra when so few heeded their first report in May 1983 of a previously unknown retrovirus that was possibly associated with AIDS.’

This is not to say that all science writing must reference Greek mythology or any other classical sources—that might soon get tedious. What it must do though, is engage, entertain and be readable. In the same way as telling you instantly which music I like best and select for my Spotify playlist, I can instantly tell you the immunology colleagues whose papers I rush to read because I know their work will be, not only important, but also, readable and enjoyable. I can also think immediately of those whose papers I dread, knowing they will be turgid and inaccessible, requiring several reads to understand what they mean (although the data may be important).

Hopefully this may inspire you to submit a piece for the Robin Weiss Prize and spread the word to others who are interested? The guidance as to the format and content are deliberately rather open-ended. Because we have centred this on single-author submissions from pre-doctoral scientists, we anticipate that submissions are less likely to comprise major datasets from wet-lab research. The submission could be a commentary or review on an area of current excitement, a personal perspective on a forthcoming challenge, a meta-analysis in a complex area, or a more technical, bioinformatics-based piece evaluating data or describing a way in which analysis could better be approached. Whichever you opt for, try and channel Robin’s spirit, and ask if your writing is accessible, beautiful and persuasive!
